# Supplementary material for: Selective production of phase-separable product from a mixture of biomass-derived aqueous oxygenates
Source: Nat Commun. 2018 Dec 5;9:5183. doi: 10.1038/s41467-018-07593-0 (PMC6281651; doi:10.1038/s41467-018-07593-0)
Supplement: Supplementary file 1 — Supplementary Information [file 41467_2018_7593_MOESM1_ESM.pdf]

Selective Production of Phase-separable Product from a Mixture of Biomass-derived Aqueous Oxygenates

Wang et al.

Supplementary Methods

Reagents

All chemicals were of analytical grade and used as purchased without further purification. Most chemicals were purchased from J&K Chemicals and Aladdin Chemicals.

Catalyst preparation

Ceria powder was prepared by a precipitation method. Briefly, 5.0 g of Ce $(NO_3)_3 \cdot 6H_2O$ was dissolved in 100 mL of Millipore-purified water (>18 MΩ·cm^{-1}, MilliQ-UV, Millipore Co., Bedford, MA), and the pH of the solution was adjusted to 11 by the addition of NH_4OH (3.4 M) under magnetic stirring at 25°C. The resulting gel mixture was washed with water, dried in an oven at 115 °C for 12 h, and calcined at 500 °C in air for 4 h. Sn doped ceria catalysts were prepared by a co-precipitation method. A $SnCl_4$ solution, $NH_3 \cdot H_2O$ and $Ce(NO_3)_3 \cdot 6H_2O$ solution were added dropwise to a big beaker simultaneously. The pH value was maintained at 11. The obtained slurry was stirred for another 4 h, after which it was centrifuged. The precipitate obtained was washed with water three times, dried at 115 °C overnight and calcined at 500 °C for 4 h. Other catalysts with nano-sized particles, including MgO, ZnO, Fe_2O_3, and SnO_2, were purchased from Aladdin Chemicals for comparison. In/Zn/Fe doped ceria catalysts were prepared by a co-precipitation method. Solutions of the corresponding metallic salts, $NH_3 \cdot H_2O$, and $Ce(NO_3)_3 \cdot 6H_2O$ solution were added to a big beaker dropwise. The pH value was maintained at 11. The obtained slurry was stirred for another 4 h, after which it was centrifuged. The precipitate obtained was washed with water three times, dried at 115 °C in an oven, and calcined at 500 °C for 4 h The content of metal oxides in doped ceria was 2% (wt/wt) relative to the ceria support.

Catalyst characterizations

Powder X-ray diffraction patterns were obtained with a Rigaku D/Max 2500/PC diffractometer, using Cu-Ka radiation at 40 kV and 20 mA. Continuous scans were collected in the 2θ range of 10-80°, at a step rate of 2°·min^{-1}. The Brunauer-Emmett-Teller (BET) surface area and pore volume were measured by nitrogen adsorption-desorption using a Quantachrome Autosorb-1. ICP analysis was conducted on an ICPS-8100 (Shimadzu). The catalyst microstructures were examined by TEM using a JEOL JEM-2000EX and a FEI Tecnai G2 F30 S-Twin. *In situ* X-ray photoelectron spectroscopy (XPS) analysis was performed using an ESCALAB250Xi (Thermo, USA), equipped with an Al-Kα (1486.6 eV) excition source. First, the catalyst was pressed into the wafers, degassed under vacuum and then placed in the analysis chamber for detection before reduction. Then, high purity H_2 (99.999%) was allowed to flow into the chamber and the chamber was heated to the set temperature for 1 h. Collection of spectra began after cooling to 25 °C under high vacuum (1×10^{-8} Pa). UV-Raman spectra were collected using a home-built spectrometer. A 325 nm constant-wave laser (Kimmon Co.) served as excitation source. The laser power was kept below 2 mW to prevent damage to the sample. A 25 mm diameter off-axis parabolic mirror (Edmund Optics Co.) served as the light collecting element. An edge filter (Semrock Co.) was used to filter Rayleigh scattered light, and a spectrograph (Shamrock 500) and UV-CCD camera (Newton 920, Andor) were used for light detection. All spectra were calibrated by placing the main Raman peak of monocrystalline Si at 520 cm^{-1}. H_2-TPR was conducted using a catalyst characterization system (Autochem 2920, Micromeritics) with a TCD detector.

Catalytic reaction in the fixed-bed reactor

The catalyst powder was pressed into pellet under 15 MPa and crushed to small particles (14-25 or 40-60 mesh). It was then loaded into a vertical reactor heated by an electric furnace. ABE or other feedstocks were fed into the reactor by a pump and N_2 was used as the carrier gas (33, 10 or 5 $mL \cdot min^{-1}$). Products and unreacted feedstock were collected in a cold trap and were analyzed by gas chromatography (GC) with tetrahydrofuran (THF) as the internal standard.

The calculations of carbon balance, 4-HPO selectivity and conversion

The carbon number in the cold-trapped liquid product was calculated using the standard curve method. The relative correction factors of feedstock and products are shown in **Supplementary Figure 2**. Taking acetone as an example, A/As is the ratio of acetone peak area to that of THF obtained from GC. The ratio of the weight of acetone to that of THF in standard solution is represented by m/ms.

The carbon number in the gaseous product was calculated using a similar method. The relative correction factors of feedstock and products with N_2 as internal standard are shown in **Supplementary Table 2**.

Supplementary Equations:

$$\text{Yield } (\%)=\frac{n(c)_{4\text{-HPO}}}{n(c)_{feed}}\times 100\% \qquad \textbf{1}$$

$$\text{Selectivity (4-HPO)}(\%)=\frac{n(c)_{4\text{-HPO}}}{n(c)_{gas}+n(c)_{liquid}-n(c)_{liquid,butanol}-n(c)_{liquid,\ ethanol}-n(c)_{liquid,\ acetone}}\times 100\% \qquad \textbf{2}$$

$$\text{Conversion}(\%)=\frac{n(c)_{feed}-n(c)_{liquid,butanol}-n(c)_{liquid,\ ethanol}-n(c)_{liquid,\ acetone}}{n(c)_{feed}}\times 100\% \qquad \textbf{3}$$

$$\text{Carbon balance } (\%)=\frac{n(c)_{gas}+n(c)_{liquid}}{n(c)_{feed}}\times 100\% \qquad \textbf{4}$$

where $n(c)_{4\text{-HPO}}$ is the number of moles of carbon atoms in 4-HPO, and $n(c)_{feed}$ is the number of moles of carbon atoms in feedstock, $n(c)_{liquid}$ is the mole of carbon atoms in the liquid trapped in the tank, including products and unreacted feedstock. $n(c)_{liquid,\ butanol}$, $n(c)_{liquid,\ acetone}$ and $n(c)_{liquid,\ ethanol}$ are the number of moles of carbon atoms of n-butanol, acetone or ethanol in liquid, respectively, which were trapped in the tank. $n(c)_{gas}$ is the number of moles of carbon atoms in gaseous products.

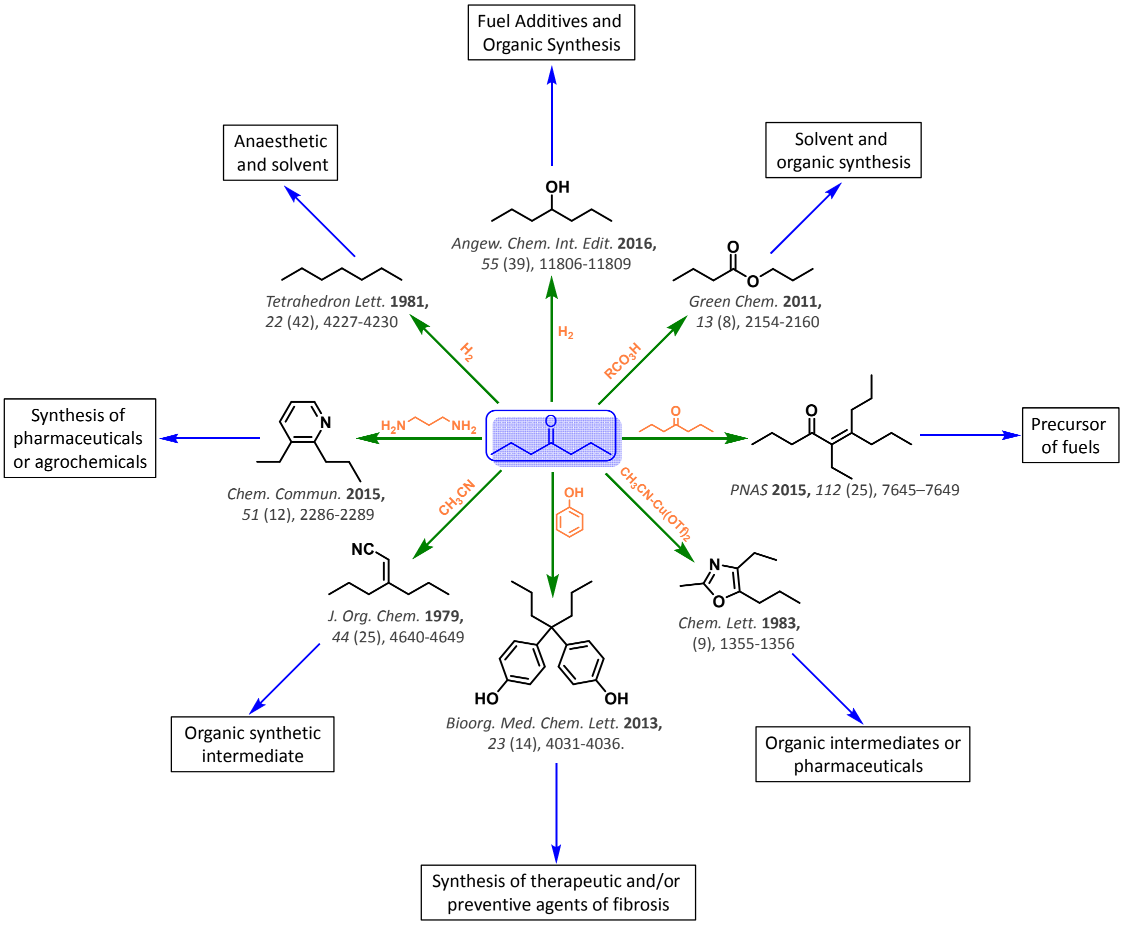

Supplementary Figure 1.

4-HPO utilization network. For example, it could be converted to C_{14} chemical, which is an important fuel additive, through self-condensation and hydrodeoxygenation. It could also be hydrogenated to 4-heptanol, which has a higher LHV (low heating value, 37.4 MJ·kg^{-1}) than *n*-butanol (33.3 MJ·kg^{-1}) and ethanol (26.8 MJ·kg^{-1}), and suitable for use as a gasoline additive (**Supplementary Table 1**).

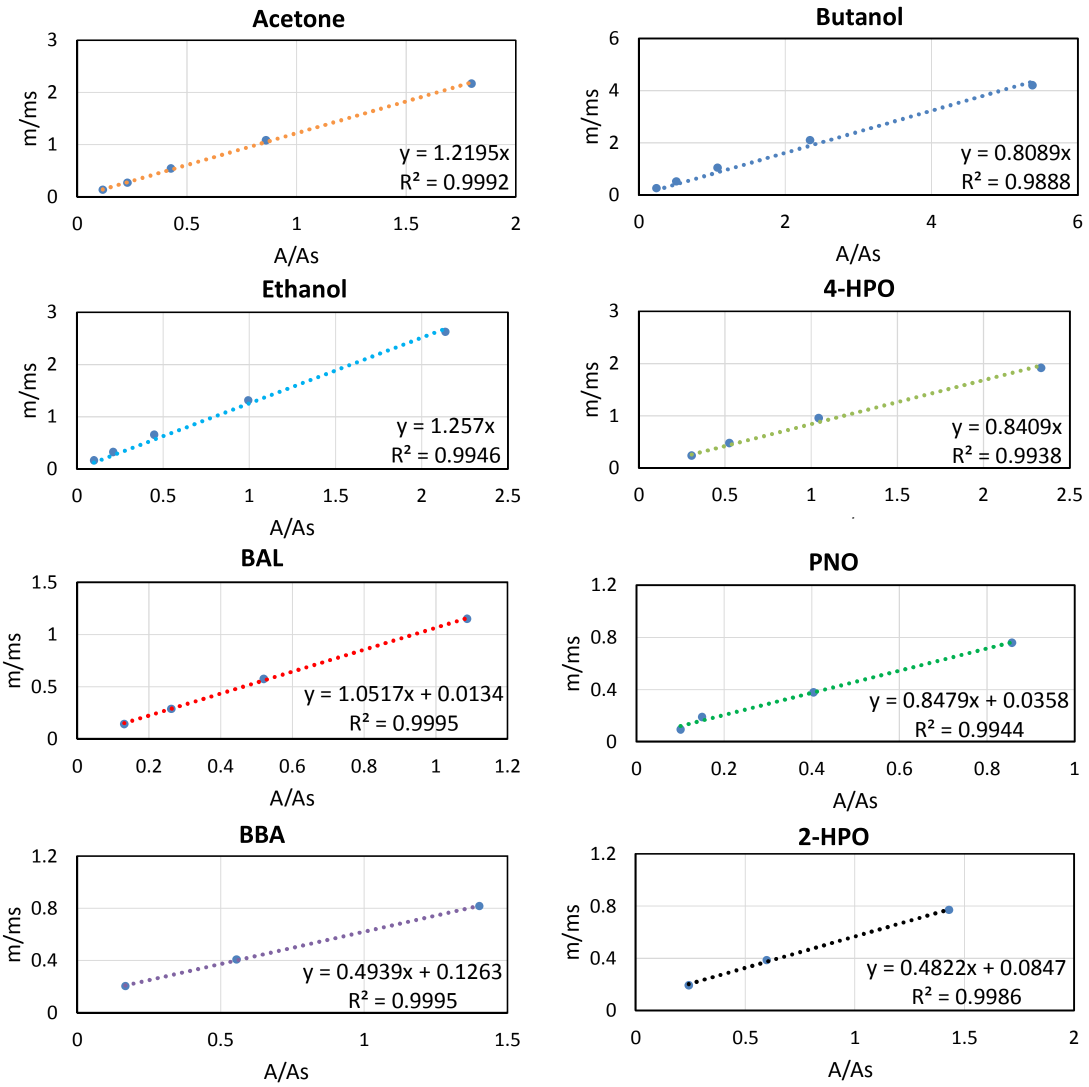

Supplementary Figure 2.

Relative correction factors for the feedstock (acetone, butanol and ethanol) and cold-trapped products, including **4-HPO** (4-heptanone), **BAL** (butanal), **PNO** (2-pentanone), **BBA** (butyl butyrate) and **2-HPO** (2-heptanone) with tetrahydrofuran (THF) as the internal standard.

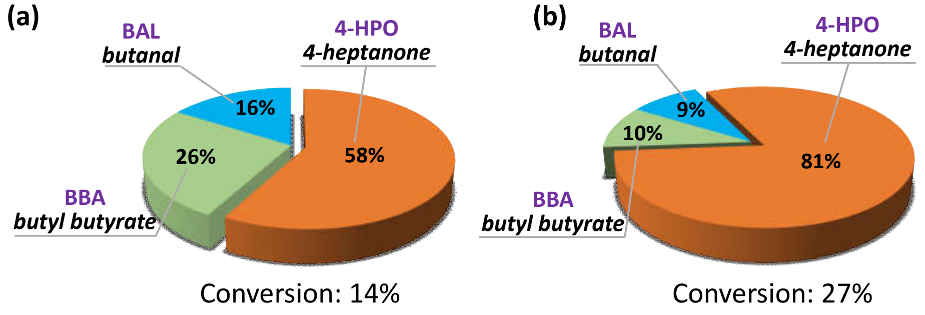

Supplementary Figure 3.

Liquid product distribution with (a) Pure *n*-butanol as the feedstock. (b) Co-feeding of *n*-butanol and water (4:1 weight ratio). Reaction conditions: ceria (16.0 g, 14-25 mesh), N_2 as carrier gas (33 mL·min^{-1}), 400 °C, WHSV= 0.5 h^{-1}.

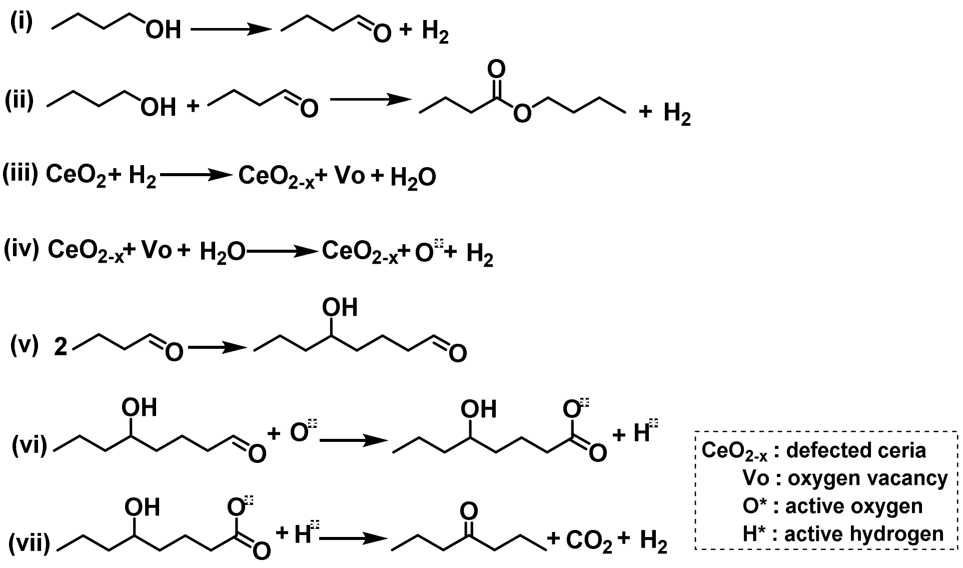

Supplementary Figure 4.
Proposed reaction steps from *n*-butanol to 4-HPO in the presence of water.

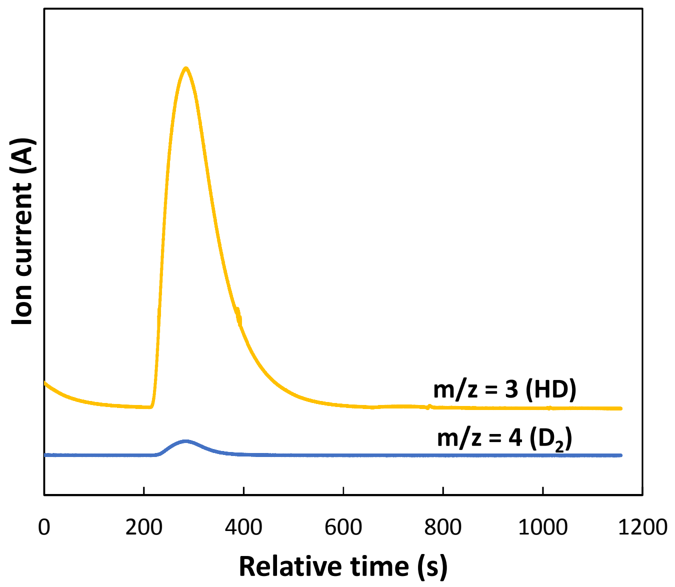

Supplementary Figure 5.
Pulse reaction with D_2O as feed (detected by mass spectroscopy) and ceria as catalyst (1 g, 14-25 mesh). 10 μL D_2O was injected at a time. H_2 (10 mL·min^{-1}) and Ar (20 mL·min^{-1}) were the carrier gases. Before detection, ceria was pretreated in H_2 for 30 min at 400 °C.

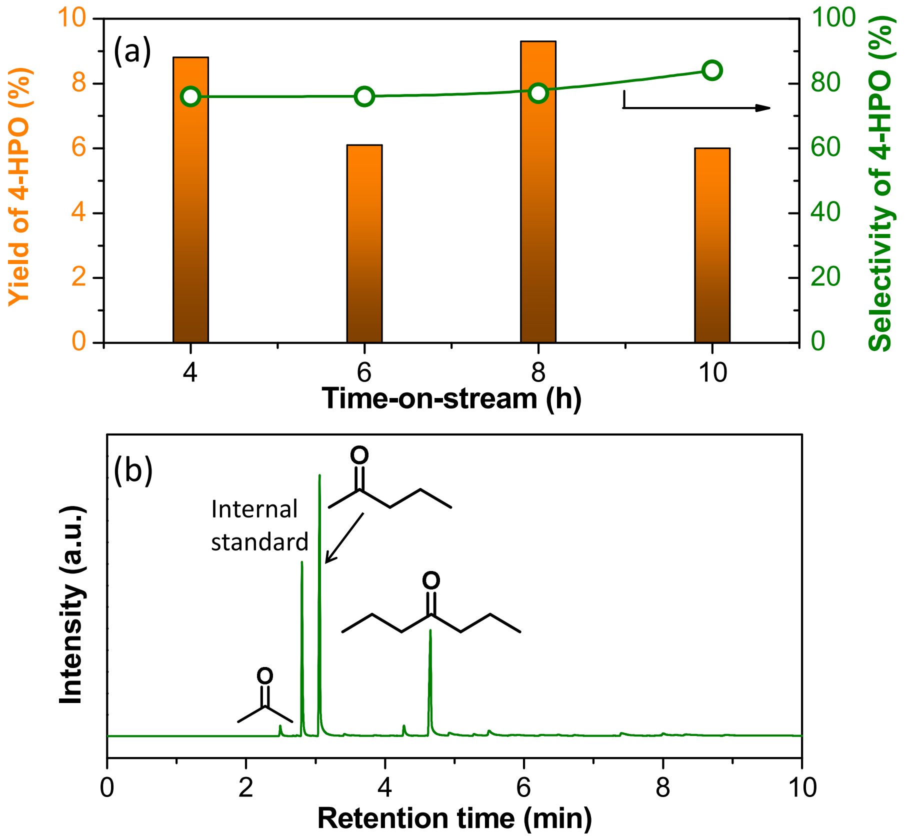

Supplementary Figure 6.

(a) Yield and selectivity of 4-HPO for the reaction with 2-pentanone and ethanol as feedstock; (b) GC profiles of liquid product trapped. Reaction conditions: ceria (3.2 g, 40-60 mesh), the mole ratio of 2-pentanone to ethanol is 1:2, N_2 as carrier gas (10 mL·min^{-1}), 420 °C, WHSV= 0.5 h^{-1}. Tetrahydrofuran is used as internal standard. Abbreviation: **4-HPO**, 4-heptanone.

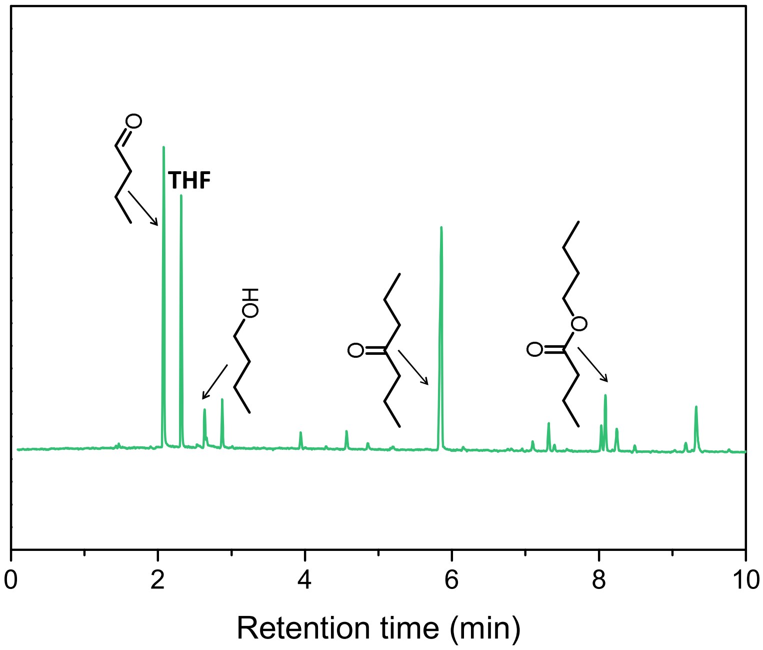

Supplementary Figure 7.

GC-MS of the liquid product from the reaction of butanal and water over ceria catalyst. Reaction conditions: catalyst, 3.2 g (40-60 mesh), butanal 0.09 mL·min^{-1}, water 0.03 mL·min^{-1}, 420 °C, N_2 as carrier gas, 10 mL·min^{-1}. The catalyst was reduced at 420 °C for 1 h in H_2 (30 ml·min^{-1}). THF was used as the internal standard.

When butanal and water were co-fed, the major product trapped in the liquid was 4-HPO with 88% selectivity (determined by GC), which is comparable to that of the reaction with butanol and water as feedstock. The conversion achieved was 83%. This suggests that the generation of 4-HPO from butanal and water is feasible.

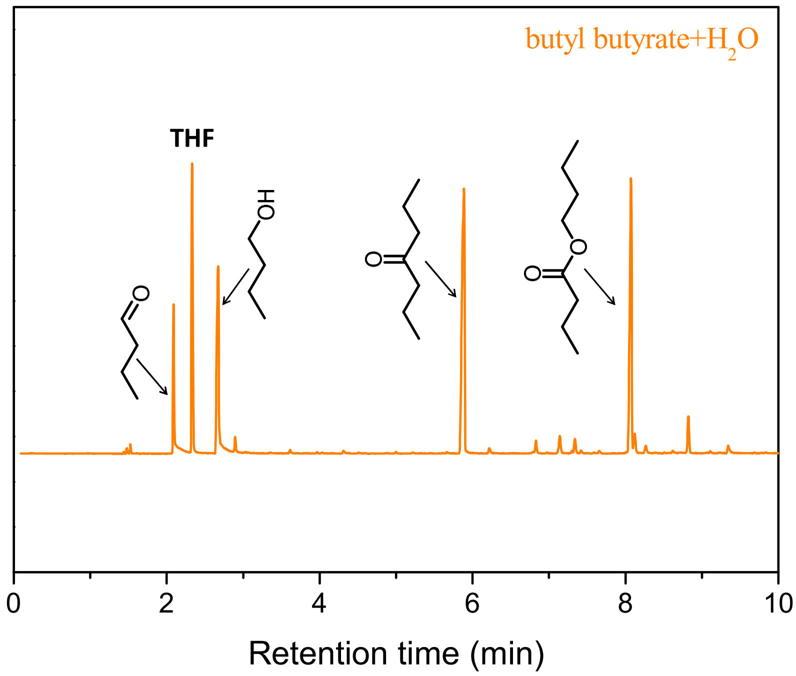

Supplementary Figure 8.

GC-MS of the liquid product from the reaction of butyl butyrate and water over ceria catalyst. Reaction conditions: catalyst, 3.2 g (40-60 mesh), butyl butyrate 0.09 mL·min^{-1}, water 0.03 mL·min^{-1}, 420 °C, N_2 as carrier gas, 10 mL·min^{-1}. The catalyst was reduced at 420 °C for 1 h in H_2 (30 ml·min^{-1}). THF was used as the internal standard.

When butyl butyrate and water were co-fed, 4-HPO is also generated with 65% selectivity in the liquid product (determined by GC), indicating the feasibility of 4-HPO generation from the reaction of butyl butyrate and water. Furthermore, butanal and butanol were generated and contributed 9% and 26% carbon numbers in the liquid products, respectively. The conversion reached 74%.

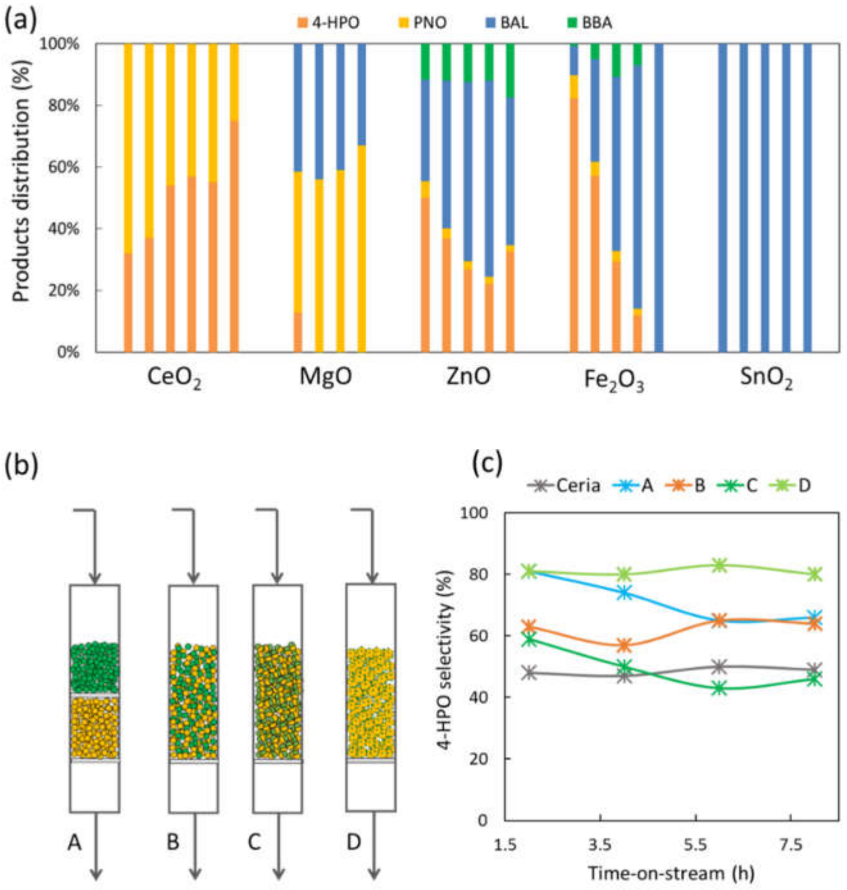

Supplementary Figure 9.

Catalytic performance of various catalysts in the aqueous ABE solution conversion reaction. (a) The distribution of products in liquid catalyzed by different metal oxides for 10 h time-on-stream. Each bar represents the product distribution of the reaction for every 2 h (ceria for 12 h and MgO for 8 h) when butanol was the feedstock. Reaction conditions: catalyst (2 g, 14-25 mesh), N_2 as carrier gas (33 mL·min^{-1}), 420 °C, feed rate of butanol (0.03 mL·min^{-1}). (b) Fixed-bed reactors were filled in different styles, marked as A, B, C, D. SnO_2 is marked using green balls, while ceria was marked using yellow balls. For Style A, SnO_2 was pressed into pellets under 15 MPa and crushed to small particles, packed on ceria and separated by a layer of inert quartz wool. For Style B, ceria and SnO_2 were pressed into pellet under 15 MPa, crushed to small particles separately, mixed uniformly, and then packed upon a layer of inert quartz wool. For Style C, ceria and SnO_2 powder were first mixed via grinding, and then pressed into pellets under 15 MPa, crushed to small particles and packed on a layer of inert quartz wool. For styles A, B and C, the weight ratio of ceria to SnO_2 was 1:1. For style D, Sn was introduced to pristine ceria via a co-precipitation method with a Sn amount of 2.0 wt%. (c) 4-HPO selectivity when ABE solution was converted over ceria, SnO_2 or Ce-Sn catalysts with four styles (A, B, C, D) described in (b). Reaction conditions: catalyst (2 g, 14-25 mesh), N_2 as carrier gas (33 mL·min^{-1}), 420 °C, WHSV=0.8 h^{-1} where A:B:E:water weight ratio is 9:51:1:22.

Abbreviations: **4-HPO**, 4-heptanone; **PNO**, 2-pentanone; **BAL**, butanal; **BBA**, butyl butyrate.

For comparison, we also tested *n*-butanol conversion over several other metal oxides, including MgO, ZnO, Fe_2O_3 and SnO_2. The product distribution in the trapped liquid is shown in **Supplementary Figure 9a**. Butanal was the major product observed in *n*-butanol conversion over these metal oxides. In particular, SnO_2 showed excellent dehydrogenation ability and afforded 99% selectivity of butanal, which is an important intermediate to 4-HPO in the presence of water. Because of the excellent dehydrogenation activity of SnO_2[1], we attempted to introduce SnO_2 to the ceria catalyst to obtain high selectivity to butanal. Thus, four styles of Sn-Ce catalytic systems (marked as A, B, C, and D) were prepared and applied to the conversion of the ABE solution (**Supplementary Figure 9b**).

For Style A, the ABE solution was converted to 4-HPO with higher selectivity (81%) initially. Then it decreased slowly and levelled off at 65%. For style B, the selectivity of 4-HPO was maintained at 65% for the entire course of the reaction, which is a result similar to that of Style A after achieving equilibrium. For Style C, the selectivity was lower than that of pristine ceria. This might be due to the formation and agglomeration of a metallic tin phase under reducing conditions at high temperature, resulting in the blockage of active sites on ceria. For Style D, the selectivity of 4-HPO was maintained above 80% for the entire course of the reaction, which indicated that doping is the best approch to introduce Sn to ceria for ABE solution conversion **(Supplementary Figure 9c)**.

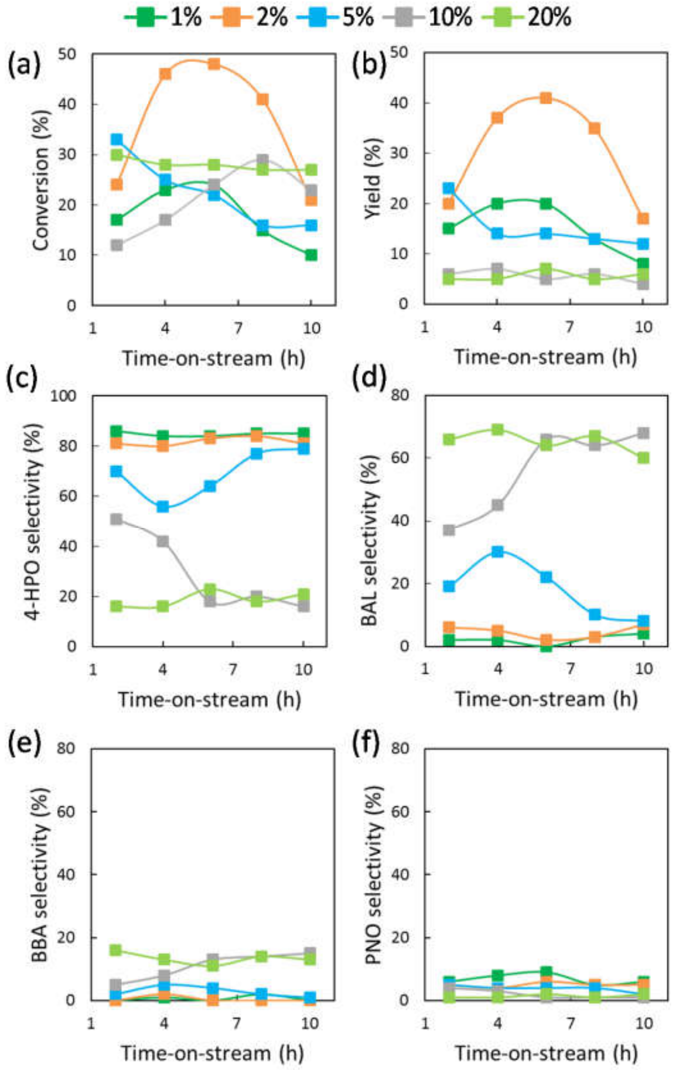

Supplementary Figure 10.

Catalytic performance on Sn-ceria catalysts with different amounts of Sn ranging from 1 wt % to 20 wt %. (a) Conversion. (b) 4-HPO yield. (c) 4-HPO selectivity. (d) BAL selectivity. (e) BBA selectivity. (f) PNO selectivity. Reaction conditions: catalyst (2 g, 40-60 mesh), N_2 as carrier gas (33 mL·min^{-1}), 420 °C, WHSV = 0.8 h^{-1}. ABE solution was used as feedstock, where the A:B:E:H_2O weight ratio was 9:51:1:22. Abbreviations: **4-HPO**, 4-heptanone; **BAL**, butanal; **BBA**, butyl butyrate; **PNO**, 2-pentanone.

The effect of the amount of doped Sn was investigated carefully. ABE solution conversion was carried out over Sn doped ceria catalysts (Sn-Ceria) with Sn amounts ranging from 1 wt% to 20 wt%. Various Sn-ceria catalysts were prepared via a co-precipitation method. The product distribution is shown in **Supplementary Figure 10**. The highest conversion (~ 48%) was obtained over 2 wt% Sn-ceria at 6 h time-on-stream (**Supplementary Figure 10a**). The 4-HPO yield increased initially and then decreased

during the course of the reaction over Sn-Ceria catalysts with lower Sn amounts (≤ 2 wt%). The highest yield (~ 41%) was obtained over 2 wt% Sn-ceria at 6 h time-on-stream (**Supplementary Figure 10b**). When the amount of Sn was low (⩽ 2 wt %), the selectivity of 4-HPO was higher than 80%. It decreased sharply with the increase in Sn loading. The selectivity dropped below 23% for 20 wt% Sn-ceria catalyst **(Supplementary Figure 10c)**. The major by-product was butanal, and its selectivity increased with the increase of Sn loading, reaching about 70% selectivity over 20 wt % Sn-ceria. This indicated the excellent dehydrogenation activity of Sn sites (**Supplementary Figure 10d**). Butyl butyrate was another remarkable by-product present with about 10% selectivity in the liquid product catalyzed by Sn-ceria catalysts with higher Sn amounts (10 wt% ~ 20 wt%) (**Supplementary Figure 10e**). The 2-pentanone selectivity was lower than 10% over various Sn-ceria catalysts (**Supplementary Figure 10f**).

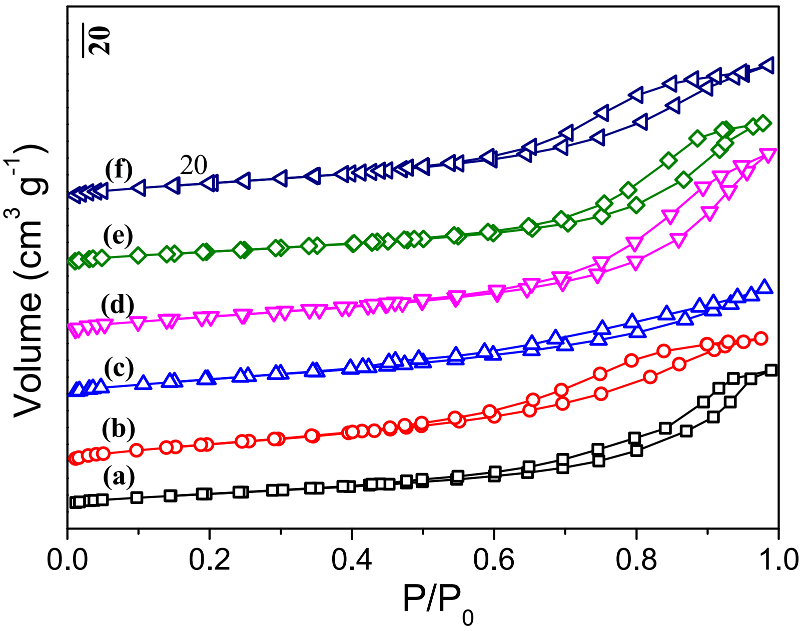

Supplementary Figure 11.

N_2 adsorption-desorption isotherms at 77 K for pristine ceria (a) and Sn-ceria catalysts with various Sn loadings (b) 1 wt%, (c) 2 wt%, (d) 5 wt%, (e) 10 wt%, (f) 20 wt%.

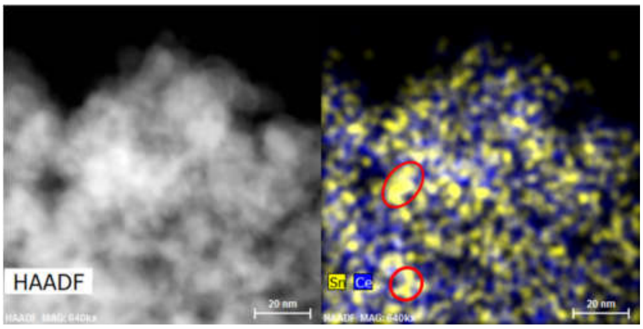

Supplementary Figure 12.

HAADF-STEM and corresponding EDS elemental mapping images of 10 wt% Sn-ceria catalyst. Particles of tin oxide were observed, which are shown in red circles. Scale bar, 20 nm.

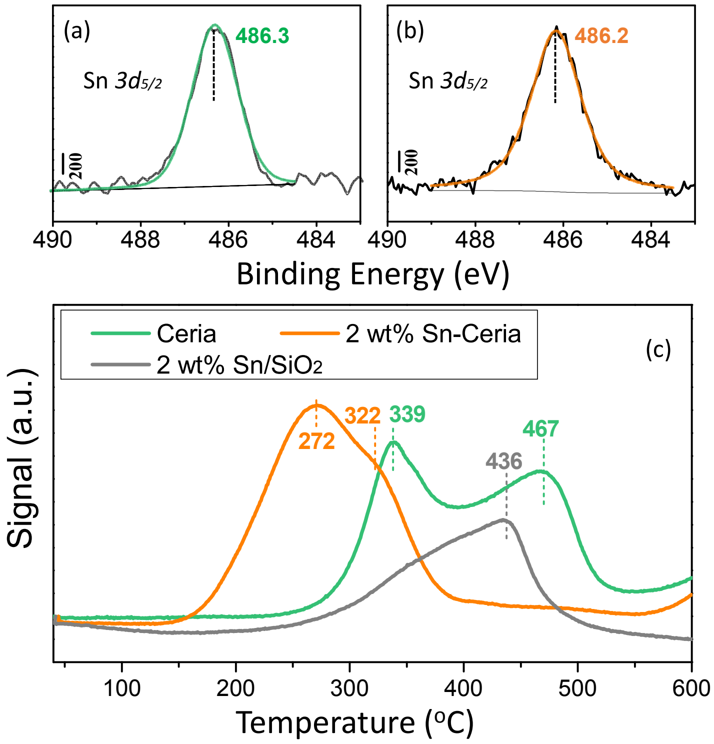

Supplementary Figure 13.

(a) Sn *$3d_{5/2}$ XPS* spectra of 2 wt% Sn-ceria (a), 2wt% Sn-ceria after hydrogen reduction at 410 °C for 1 h (b), and H_2-TPR profiles of ceria, 2 wt% Sn/SiO_2 and 2 wt% Sn-ceria catalysts.

The valence states of Sn were investigated by *in situ XPS* measurements (**Supplementary Figure 13a**). For 2 wt% Sn-ceria catalyst, a peak was resolved at 486.3 eV. This peak is assigned to the Sn (IV), although the value was lower than those reported previously.[2] The Sn (IV) species is present even after reduction at 410 °C in H_2 (**Supplementary Figure 13b**). This suggests that the unique properties of ceria as matrix prevent the reduction of Sn(IV) to a lower charge state, which is in a good agreement with the results of H_2-TPR (**Supplementary Figure 13c**). The peaks at 272 °C and 322 °C in the H_2-TPR of fresh Sn-ceria catalyst, which were located at lower temperatures compared with pristine ceria (339 °C and 467 °C, respectively), were caused by the removal of the surface oxygen of the ceria matrix, indicating that the addition of Sn promoted the reduction of ceria.[3] The feasible reduction for fresh Sn-ceria catalyst at lower temperatures leads to the generation of a large number of oxygen vacancies during reduction. There is no obvious peak assigned to the reduction of SnO_2 for fresh Sn-ceria catalyst before 410 °C. In contrast, for SnO_2 supported on SiO_2, the reduction of SnO_2 was at temperatures as low as 250 °C.

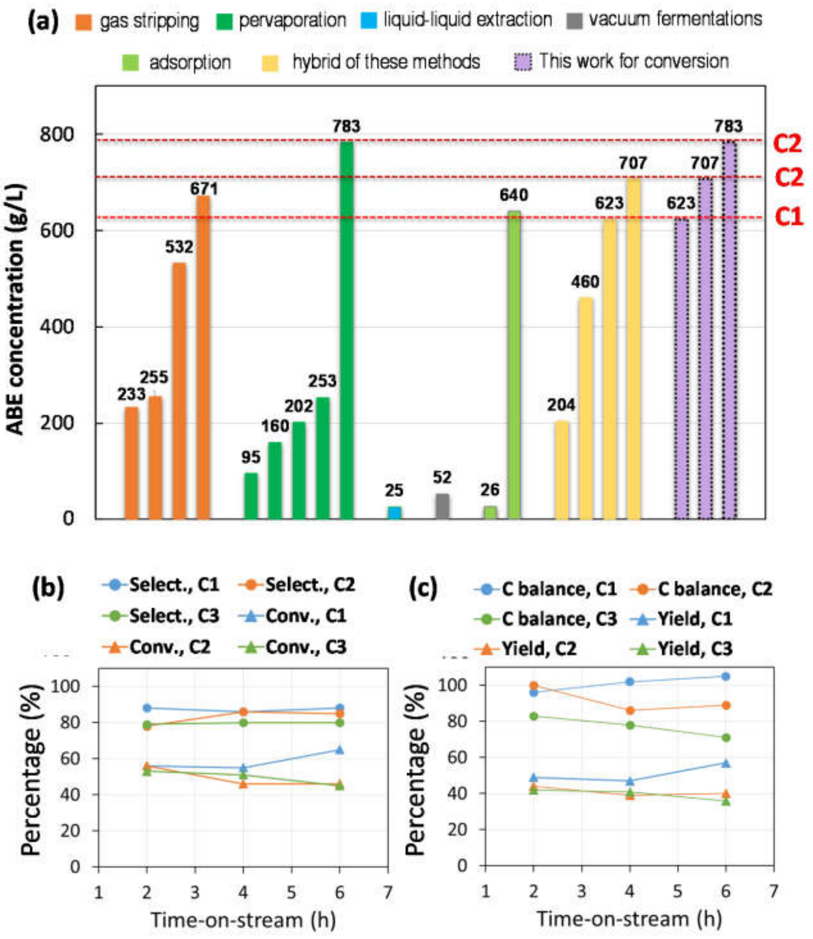

Supplementary Figure 14.

Effect of water content on the aqueous ABE solution conversion reaction. (a) ABE concentration after different methods of recovery previously reported. (b) Conversion and 4-HPO selectivity, and (c) carbon balance and 4-HPO yield in aqueous ABE solution conversion reactions with different ABE concentrations (623 $g{\cdot}L^{-1}$, 707 $g{\cdot}L^{-1}$, and 783 $g{\cdot}L^{-1}$; marked as C1, C2 and C3 respectively), where A:B:E weight ratio is 9:51:1. Reaction conditions: 3.2 g 2 wt% Sn-ceria, 0.03 $mL{\cdot}min^{-1}$, N_2 as carrier gas (10 $mL{\cdot}min^{-1}$). These reactions were conducted at 420 °C.

On the basis of the ABE recovery development in ABE fermentation[4-20] (**Supplementary Figure 14a**), we prepared various ABE solutions with concentrations of 623 g/L, 707 g/L, and 783 g/L, with corresponding water content of 27 wt%, 15 wt% and 3.5 wt%, where A:B:E=9:51:1. These three ABE solutions were then used as feedstocks for reaction over 2 wt% Sn-ceria catalyst. The results suggested that water content ranging from 3.5 wt% to 27 wt% had no apparent effect on 4-HPO selectivity, which remains at 80%-88%. However, larger amounts of water are beneficial to ABE conversion, resulting in 50 %-57% conversions (**Supplementary Figure 14b**).

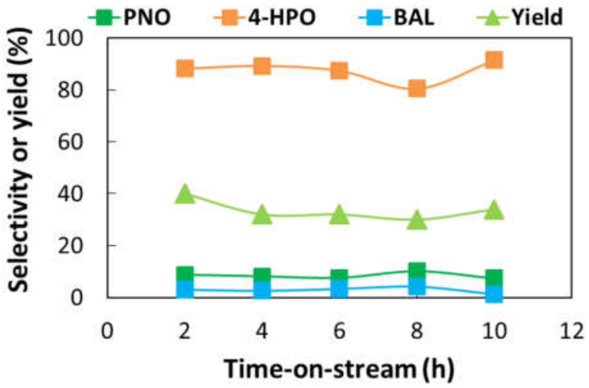

Supplementary Figure 15.

The selectivity and yield of 4-HPO over 2 wt% Sn-ceria catalyst with ABE solution as the feedstock with the addition of organic acids. 72 mL ABE solution as feedstock (A:B:E:H_2O weight ratio is 9:51:1:22) with the addition of 1 mL acetic acid and 1 mL butyric acid. Abbreviations: **4-HPO**, 4-heptanone; **PNO**, 2-pentanone; **BAL**, butanal; **2-HPO**, 2-heptanone.

Organic acids, primarily acetic acid and butyric acid, are usually produced during the ABE fermentation process.[21,22] Small amounts of these two acids (1.7 wt% acetic acid and 1.5 wt% butyric acid) were added to a synthetic ABE broth and co-fed over 2 wt% Sn-ceria catalyst at 420 °C (**Supplementary Figure 15**). The addition of these acids did not generate new products. Conversely, the 4-HPO selectivity was augmented to 90%. This might be due to the following two reasons: (1) the ketonization of butyric acid catalyzed by ceria-based catalysts generates 4-HPO;[23,24] (2) the ketonization of acetic acid forms acetone, which reacts with acetaldehyde to form 2-pentanone and then forms 4-HPO [25,26].

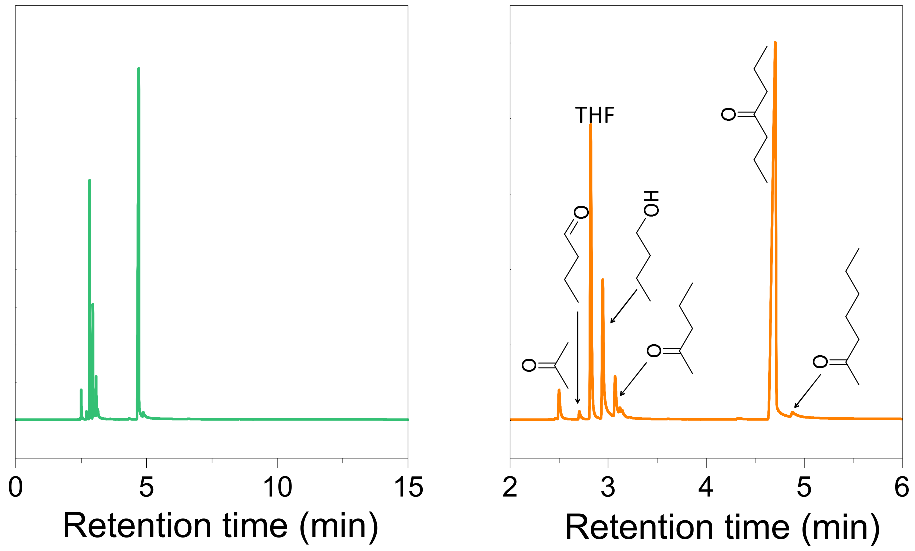

Supplementary Figure 16.

A typical GC result of ABE conversion over 2 wt% Sn-ceria during the long time test. THF was used as the internal standard. The conversion here is 71% with 84% 4-HPO selectivity.

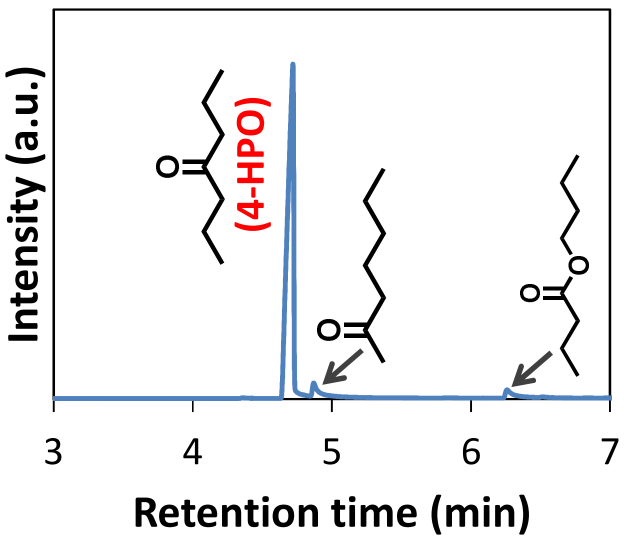

Supplementary Figure 17.
GC profile of 4-HPO with 95% purity separated from the liquid products during the continuous reaction via simple distillation.

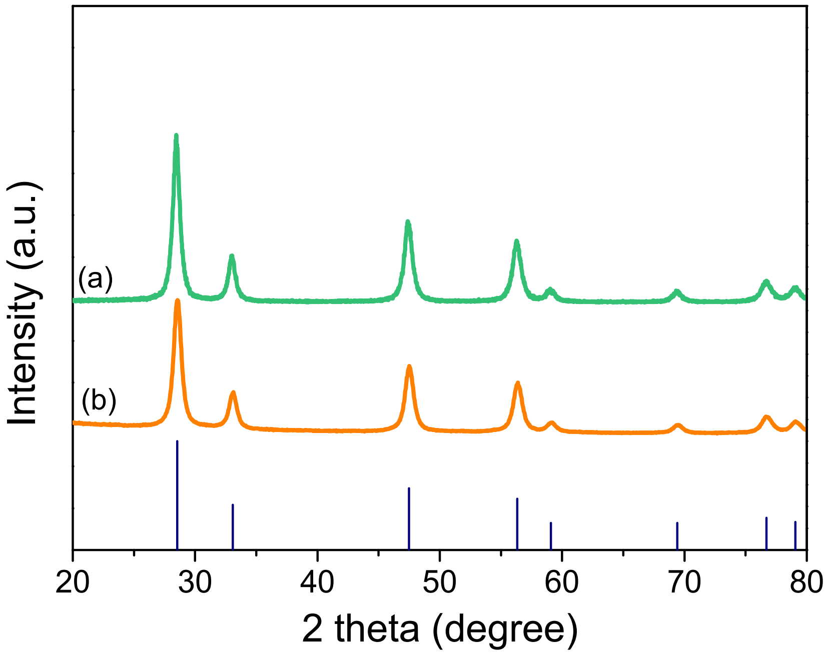

Supplementary Figure 18.

X-ray diffraction patterns (using Cu Kα radiation) of 2 wt% Sn-ceria (a) and spent 2 wt% Sn-ceria (b) catalysts. The spent 2 wt% Sn-ceria is a recycled catalyst sample after reaction for 120 h.

All the diffractions correspond to the pure phase ceria. The crystallite size, which is around 11 nm (determined by XRD) shows no remarkable change after the reaction.

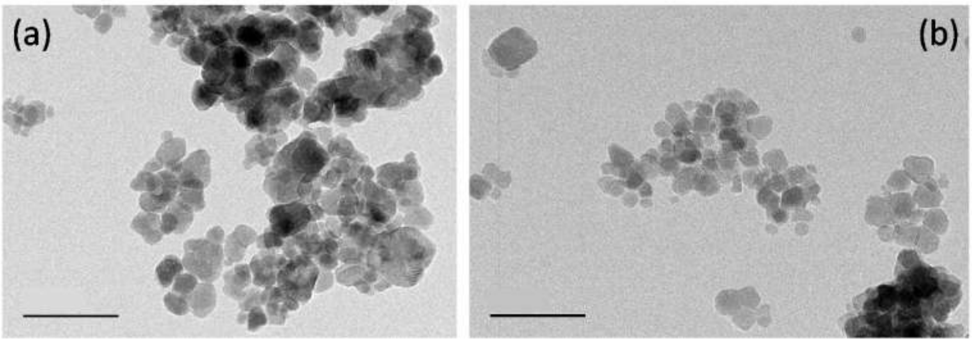

Supplementary Figure 19.

TEM of fresh 2 wt% Sn-ceria (a) and spent 2 wt% Sn-ceria (b) catalysts. The spent 2 wt% Sn-ceria is a recycled catalyst sample after reaction for 120 h. Scale bar, 50 nm.

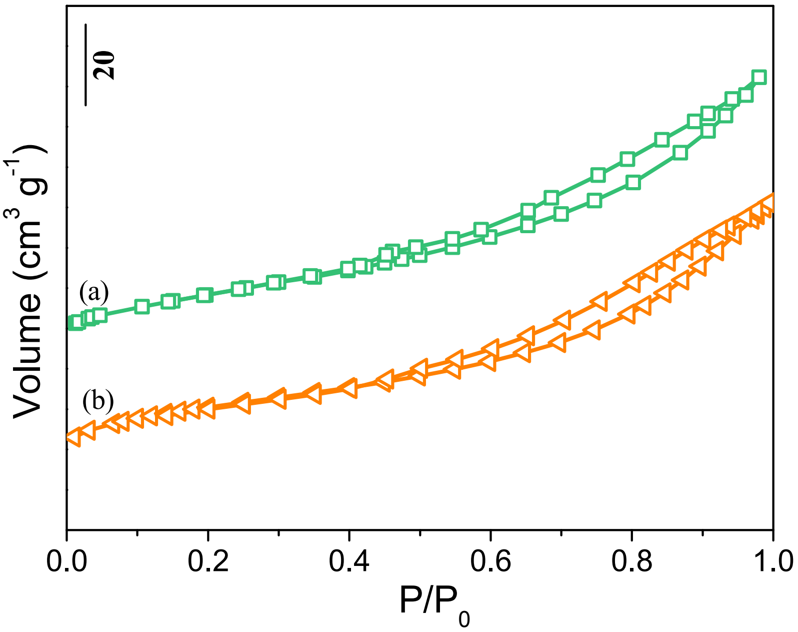

Supplementary Figure 20.

N_2 adsorption-desorption isotherms at 77 K of 2 wt% Sn-ceria (a) and spent 2 wt% Sn-ceria (b) catalysts. The spent 2 wt% Sn-ceria is a recycled catalyst sample after reaction for 120 h.

The specific surface area decreases from 66.3 $m^2 \cdot g^{-1}$ to 49.0 $m^2 \cdot g^{-1}$, while the pore volume decreases from 0.11 $cm^3 \cdot g^{-1}$ to 0.09 $cm^3 \cdot g^{-1}$, which may be due to the carbonaceous deposition over the pore structure.

Supplementary Table 1.

Comparison of ethanol, *n*-butanol, and 4-heptanol

	LHV ($MJ \cdot kg^{-1}$)	Boiling point (°C)	Oxygen content (%)
**Ethanol**	26.8	78	34.8
**n-Butanol**	33.3	118	22.0
**4-Heptanol**	37.4[a]	156	13.8

[a] LHV of 4-heptanol was caution method of Benson.

Supplementary Table 2.

Results of the relative correction factors for gas products, including CO, CO_2, and CH_4.

	Retention time	Peak area	Gas rate
N_2	1.036	370834	10 ml min^{-1}
CO_2	5.719	70988	1 ml min^{-1}

$$\frac{V(CO_2)}{V(N_2)} = \boldsymbol{f(CO_2)} \frac{A(CO_2)}{A(N_2)} \qquad \boldsymbol{f(CO_2)} = 0.522$$

	Retention time	Peak area	Gas rate
N_2	1.011	369981	10 ml min^{-1}
CH_4	2.976	61479	1 ml min^{-1}

$$\frac{V(CH_4)}{V(N_2)} = \boldsymbol{f(CH_4)} \frac{A(CH_4)}{A(N_2)} \qquad \boldsymbol{f(CH_4)} = 0.602$$

	Retention time	Peak area	Gas rate
N_2	1.019	347855	10 ml min^{-1}
CO	1.302	95717	1 ml min^{-1}

$$\frac{V(CO)}{V(N_2)} = \boldsymbol{f(CO)} \frac{A(CO)}{A(N_2)} \qquad \boldsymbol{f(CO)} = 0.363$$

Supplementary Table 3.

Sn loading of Sn-ceria catalysts calculated based on SnO_2 by ICP

Catalysts	SnO_2 loading by ICP (wt%)
**1 wt% Sn-ceria**	1.1
**2 wt% Sn-ceria**	2.3
**5 wt% Sn-ceria**	5.5
**10 wt% Sn-ceria**	7.6
**20 wt% Sn-ceria**	5.4

Supplementary Table 4.

Crystallite sizes and ceria crystallinity of ceria and Sn doped ceria catalysts.

Catalyst	crystallite size (nm)	Crystallinity (%)
**Ceria**	11	58.2
**1 wt% Sn-ceria**	13	60.2
**2 wt% Sn-ceria**	11	57.9
**5 wt% Sn-ceria**	11	60.6
**10 wt% Sn-ceria**	12	59.0
**20 wt% Sn-ceria**	11	62.1

Supplementary Table 5.

BET surface area and pore volume of pristine ceria and Sn-ceria

Catalysts	BET ($m^2 \cdot g^{-1}$)	Pore volume ($cm^3 \cdot g^{-1}$)
**Ceria**	49.8	0.14
**1 wt% Sn-ceria**	75.8	0.13
**2 wt% Sn-ceria**	66.3	0.11
**5 wt% Sn-ceria**	62.5	0.18
**10 wt% Sn-ceria**	49.2	0.14
**20 wt% Sn-ceria**	60.3	0.13

Supplementary References:

1 Barias, O. A., Holmen, A. & Blekkan, E. A. Propane dehydrogenation over supported Pt and Pt-Sn catalysts: Catalyst preparation, characterization, and activity measurements. *J. Catal.* **158**, 1-12 (1996).

2 Merlen, E. et al. Characterization of bimetallic Pt-Sn/Al2O3 catalysts: Relationship between particle size and structure. *J. Catal.* **159**, 178-188 (1996).

3 An, J. H. et al. Acid-Promoter-Free Ethylene Methoxycarbonylation over Ru-Clusters/Ceria: The Catalysis of Interfacial Lewis Acid-Base Pair. *J. Am. Chem. Soc.* **140**, 4172-4181 (2018).

4 Cai, D. et al. Acetone-butanol-ethanol (ABE) fermentation integrated with simplified gas stripping using sweet sorghum bagasse as immobilized carrier. *Chem. Eng. J.* **277**, 176-185 (2015).

5 Ezeji, T. C., Qureshi, N. & Blaschek, H. P. Acetone butanol ethanol (ABE) production from concentrated substrate: reduction in substrate inhibition by fed-batch technique and product inhibition by gas stripping. *Appl. Microbiol. Biotechnol.* **63**, 653-658 (2004).

6 Li, J. et al. Efficient production of acetone-butanol-ethanol (ABE) from cassava by a fermentation-pervaporation coupled process. *Bioresour. Technol.* **169**, 251-257 (2014).

7 Xue, C. et al. Evaluation of hydrophobic micro-zeolite-mixed matrix membrane and integrated with acetone-butanol-ethanol fermentation for enhanced butanol production. *Biotechnol Biofuels* **8**, 105 (2015).

8 Kong, X. P. et al. Efficient acetone-butanol-ethanol (ABE) production by a butanol-tolerant mutant of Clostridium beijerinckii in a fermentation-pervaporation coupled process. *Biochem. Eng. J.* **105**, 90-96 (2016).

9 Li, J. et al. Continuous Acetone-Butanol-Ethanol (ABE) Fermentation with in Situ Solvent Recovery by Silicalite-1 Filled PDMS/PAN Composite Membrane. *Energ. Fuel.* **28**, 555-562 (2014).

10 Lee, S. H. et al. Ex situ product recovery for enhanced butanol production by Clostridium beijerinckii. *Bioprocess Biosystems Eng.* **39**, 695-702 (2016).

11 Xue, C. et al. Butanol production in acetone-butanol-ethanol fermentation with in situ product recovery by adsorption. *Bioresour. Technol.* **219**, 158-168 (2016).

12 Lu, K. M., Chiang, Y. S., Wang, Y. R., Chein, R. Y. & Li, S. Y. Performance of fed-batch acetone-butanol-ethanol (ABE) fermentation coupled with the integrated in situ extraction-gas stripping process and the fractional condensation. *J Taiwan Inst Chem E* **60**, 119-123 (2016).

13 Lu, K. M. & Li, S. Y. An integrated in situ extraction-gas stripping process for Acetone-Butanol-Ethanol (ABE) fermentation. *J Taiwan Inst Chem E* **45**, 2106-2110 (2014).

14 Xue, C. et al. Two-stage in situ gas stripping for enhanced butanol fermentation and energy-saving product recovery. *Bioresour. Technol.* **135**, 396-402 (2013).

15 Xue, C. et al. Characterization of gas stripping and its integration with acetone-butanol-ethanol fermentation for high-efficient butanol production and recovery. *Biochem. Eng. J.* **83**, 55-61 (2014).

16 Cai, D. et al. Gas stripping-pervaporation hybrid process for energy-saving product recovery from acetone-butanol-ethanol (ABE) fermentation broth. *Chem. Eng. J.* **287**, 1-10 (2016).

17 Xue, C. et al. A novel in situ gas stripping-pervaporation process integrated with acetone-butanol-ethanol fermentation for hyper n-butanol production. *Biotechnol. Bioeng.* **113**, 120-129 (2016).

18 Cai, D. et al. Two-stage pervaporation process for effective in situ removal acetone-butanol-ethanol from fermentation broth. *Bioresour. Technol.* **224**, 380-388 (2017).

19 Bankar, S. B., Survase, S. A., Singhal, R. S. & Granstrom, T. Continuous two stage acetone-butanol-ethanol fermentation with integrated solvent removal using Clostridium acetobutylicum B 5313. *Bioresour. Technol.* **106**, 110-116 (2012).

20 Zhou, H. L., Su, Y. & Wan, Y. H. Phase separation of an acetone-butanol-ethanol (ABE)-water mixture in the permeate during pervaporation of a dilute ABE solution. *Sep. Purif. Technol.* **132**, 354-361 (2014).

21 Thang, V. H., Kanda, K. & Kobayashi, G. Production of acetone-butanol-ethanol (ABE) in direct fermentation of cassava by Clostridium saccharoperbutylacetonicum N1-4. *Appl. Biochem. Biotechnol.* **161**, 157-170 (2010).

22 Kujawska, A., Kujawski, J., Bryjak, M. & Kujawski, W. ABE fermentation products recovery methods-A review. *Renew. Sust. Energ. Rev.* **48**, 648-661 (2015).

23 Murkute, A. D., Jackson, J. E. & Miller, D. J. Supported mesoporous solid base catalysts for condensation of carboxylic acids. *J. Catal.* **278**, 189-199 (2011).

24 Nagashima, O., Sato, S., Takahashi, R. & Sodesawa, T. Ketonization of carboxylic acids over CeO2-based composite oxides. *J. Mol. Catal. A-Chem.* **227**, 231-239 (2005).
25 Snell, R. W. & Shanks, B. H. Ceria calcination temperature influence on acetic acid ketonization: Mechanistic insights. *Appl. Catal. A-Gen.* **451**, 86-93 (2013).
26 Hasan, M. A., Zaki, M. I. & Pasupulety, L. Oxide-catalyzed conversion of acetic acid into acetone: an FTIR spectroscopic investigation. *Appl. Catal. A-Gen.* **243**, 81-92 (2003).
